# Comparison of SARS-CoV-2 spike-specific IgA and IgG in nasal secretions, saliva and serum

**DOI:** 10.3389/fimmu.2024.1346749

**Published:** 2024-03-15

**Authors:** Oscar Bladh, Katherina Aguilera, Ulrika Marking, Martha Kihlgren, Nina Greilert Norin, Anna Smed-Sörensen, Margaret Sällberg Chen, Jonas Klingström, Kim Blom, Michael W. Russell, Sebastian Havervall, Charlotte Thålin, Mikael Åberg

**Affiliations:** ^1^ Department of Clinical Sciences, Danderyd Hospital, Karolinska Institutet, Stockholm, Sweden; ^2^ Public Health Agency of Sweden, Solna, Sweden; ^3^ Division of Immunology and Allergy, Department of Medicine Solna, Karolinska Institutet, Karolinska University Hospital, Stockholm, Sweden; ^4^ Department of Dental Medicine, Karolinska Institutet, Stockholm, Sweden; ^5^ Division of Pathology, Department of Laboratory Medicine, Karolinska Institutet, Stockholm, Sweden; ^6^ Department of Biomedical and Clinical Sciences (BKV), Linköping University, Linköping, Sweden; ^7^ Department of Microbiology and Immunology, Jacobs School of Medicine and Biomedical Sciences, University at Buffalo, Buffalo, NY, United States; ^8^ Department of Medical Sciences, Clinical Chemistry and SciLifeLab Affinity Proteomics, Uppsala University, Uppsala, Sweden

**Keywords:** SARS-CoV-2, Covid-19, vaccines, mucosal immunity, antibodies, secretory IgA, saliva sampling, nasal sampling

## Abstract

**Introduction:**

Several novel vaccine platforms aim at mucosal immunity in the respiratory tract to block SARS-CoV-2 transmission. Standardized methods for mucosal sample collection and quantification of mucosal antibodies are therefore urgently needed for harmonized comparisons and interpretations across mucosal vaccine trials and real-world data.

**Methods:**

Using commercial electrochemiluminescence antibody panels, we compared SARS-CoV-2 spike-specific IgA and IgG in paired saliva, nasal secretions, and serum from 1048 healthcare workers with and without prior infection.

**Results:**

Spike-specific IgA correlated well in nasal secretions and saliva (r>0.65, p<0.0001), but the levels were more than three-fold higher in nasal secretions as compared to in saliva (p<0.01). Correlations between the total population of spike-specific IgA and spike-specific secretory IgA (SIgA) were significantly stronger (p<0.0001) in nasal secretions (r=0.96, p<0.0001) as opposed to in saliva (r=0.77, p<0.0001), and spike-specific IgA correlated stronger (p<0.0001) between serum and saliva (r=0.73, p<0.001) as opposed to between serum and nasal secretions (r=0.54, p<0.001), suggesting transudation of monomeric spike specific IgA from the circulation to saliva. Notably, spike-specific SIgA had a markedly higher SARS-CoV-2 variant cross-binding capacity as compared to the total population of spike specific IgA and IgG in both nasal secretions, saliva and serum, (all p<0.0001), which emphasizes the importance of taking potential serum derived monomeric IgA into consideration when investigating mucosal immune responses.

**Discussion:**

Taken together, although spike-specific IgA can be reliably measured in both nasal secretions and saliva, our findings imply an advantage of higher levels and likely also a larger proportion of SIgA in nasal secretions as compared to in saliva. We further corroborate the superior variant cross-binding capacity of SIgA in mucosal secretions, highlighting the potential protective benefits of a vaccine targeting the upper respiratory tract.

## Introduction

Current intramuscular SARS-CoV-2 vaccines protect against severe disease and death (1, 2), but do not prevent breakthrough infections with recent omicron variants (3, 4). The continuous viral transmission has triggered a rapid development of novel vaccine platforms aiming to raise robust mucosal immunity in the respiratory tract, either alone or in combination with an intramuscular vaccine (5, 6). However, a critical gap remains in our understanding of mucosal immune responses to SARS-CoV-2, particularly in how these responses are elicited, and maintained. Moreover, standardized methods for the collection and quantification of mucosal antibody responses are currently lacking. Establishing a standardization of these methods would allow for harmonized comparisons and interpretations across clinical trials and real-world data.

The primary antibody isotype in mucosal secretions is IgA (7) produced by plasma cells residing beneath the epithelial layer of the mucosa (8, 9). While the circulating form of IgA is mainly monomeric, respiratory mucosal secretions predominantly contain polymeric IgA, i.e., 2 or more monomeric IgA units (mainly dimers and tetramers) linked by a joining (J) chain.

(10, 11). The dimeric form exhibits superior virus-neutralizing capacity compared to IgG and monomeric IgA (12, 13). Dimeric IgA bind to the polymeric immunoglobulin (pIg) receptor on the basal surface of mucosal epithelial cells, initiating the transport of dimeric IgA across the epithelium and the release of secretory IgA (SIgA) into mucosal secretions (14, 15). SIgA is resistant to a broad range of proteases originating from both the host and microbes (16, 17). Currently, however, most IgA immune assays do not distinguish between monomeric, polymeric or SIgA.

Mucosal SARS-CoV-2 spike-specific IgA is predominantly detected in individuals with prior SARS-CoV-2 infection (18–23) and can be detected for up to nine months post infection (19, 24). The different collection methods and localizations for respiratory mucosal sampling used in these studies illustrate the complexity to compare findings in mucosal immunology research. Samples from the nasal cavity can easily be collected by swabbing the lining of the anterior nostrils (18–20), or by nasosorption (24), while saliva samples can be collected by passive drooling (25), or various saliva collection devices such as absorbent cotton rolls or cotton swabs (21). Passive drooling confers the least interference with the oral mucosa, renders relatively large saliva volumes and is the gold standard for saliva collection. However, passive drooling is a relatively time-consuming method and can be logistically challenging due to its cumbersome nature. Collecting saliva with absorbent cotton rolls kept in the mouth, with or without chewing, could circumvent these issues but may skew results by stimulating saliva production. Alternatively, buccal swabbing is a rapid approach with minimal interference with the oral mucosa and low risk of saliva exposure to healthcare professionals but may not be suitable for investigations where large volumes of saliva are needed.

Taken together, choice of collection technique and sampling localization could introduce variations and confounding factors, and to our knowledge there is yet no head-to-head comparison of SARS-CoV-2 Ig in saliva and nasal secretions. As a first measure to standardize mucosal sampling, this study therefore compared SARS-CoV-2 spike-specific IgA and IgG in 1048 paired saliva, nasal secretion and serum samples from healthcare workers (HCW) enrolled in the COMMUNITY (Covid-19 Biomarker and Immunity) cohort-study (18–20, 23, 26–37).

## Methods

### Study population

The ongoing COMMUNITY cohort study investigates immune responses to SARS-CoV-2 infection and vaccination. 2149 HCW were enrolled at Danderyd Hospital, Stockholm, Sweden, in April 2020. Study participants have since enrollment been followed-up every four months. Serum, saliva and nasal secretion samples are collected at each follow-up. Vaccination data (type and time) is obtained from the Swedish vaccination register (VAL Vaccinera). SARS-CoV-2 infection is defined as either seroconversion to the SARS-CoV-2 spike antigen at any of the follow-ups prior to vaccination, seroconversion to the SARS-CoV-2 nucleocapsid antigen, a record of a positive qPCR test in the national communicable diseases register or a positive Rapid Diagnostic Test reported at any of the follow-ups within the study.

**Figure 1:**
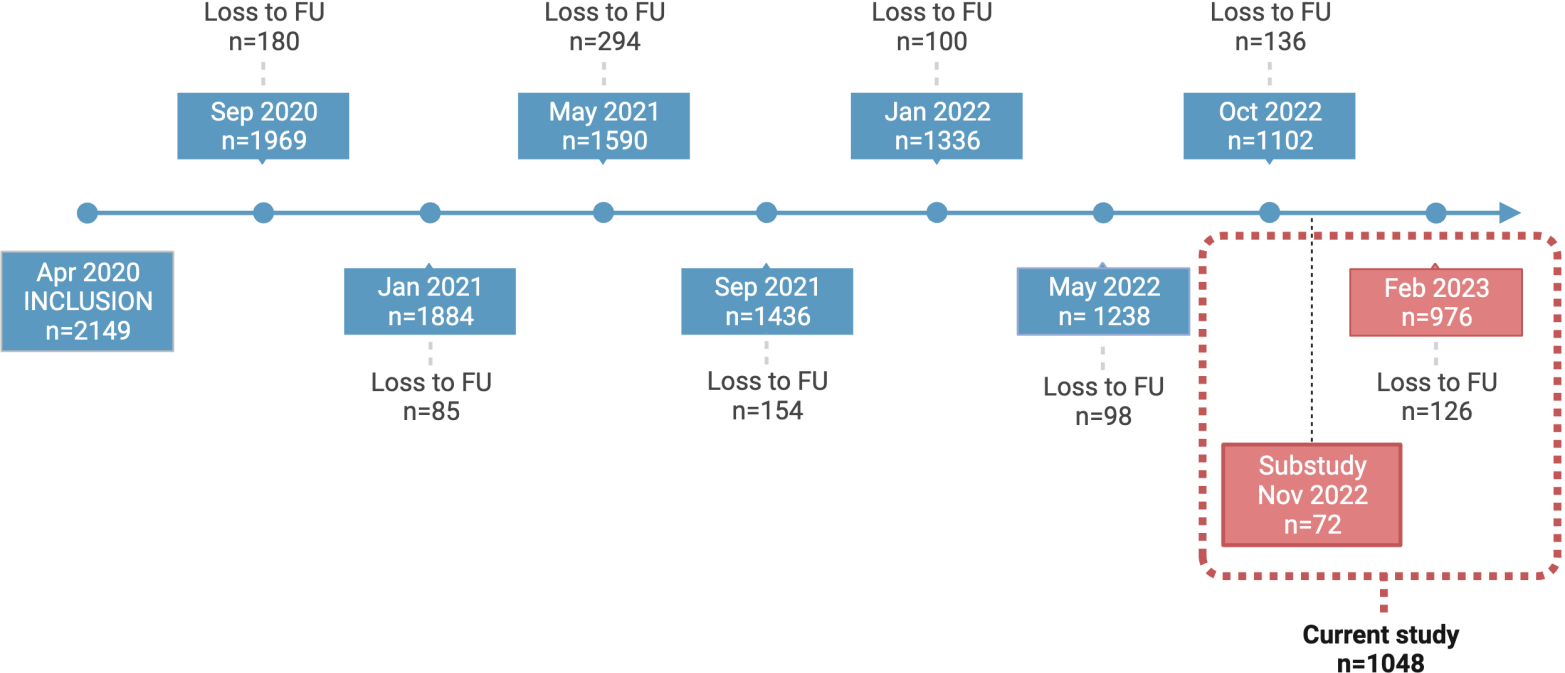
COMMUNITY cohort study timeline. Study participants in the COMMUNITY cohort (n=2149) were initially enrolled in April 2020 and have been followed regularly since then. This figure depicts all regular samplings in addition to the specific follow-up designed for this sub study. Participants enrolled in this sub study (n=1048) were sampled in November 2022 (n=72) and February 2023 (n=976). FU, Follow-up.

For this sub study, serum, saliva and nasal secretion samples were obtained from 72 HCW in November 2022 (**Table 1**; **Figure 1**), and from 976 HCW in February 2023 (**Table 2**; **Figure 1**). Participants were stratified according to prior SARS-CoV-2 infection. Written informed consent was obtained from all study participants and the study protocol was approved by the Swedish Ethical Review Authority (dnr 2020-01653).

**Table 1:** Demographics of 72 healthcare workers sampled November 2022.

	1-3 prior infections (N=61)	No prior infection (N=11)	Overall (N=72)
Age			
Median [Min, Max]	55 [34, 72]	53 [40, 71]	55 [34, 72]
Sex			
Female	52 (85.2%)	11 (100%)	63 (87.5%)
Male	9 (14.8%)	0 (0%)	9 (12.5%)
Vaccine doses			
No Vaccine	1 (1.6%)	0 (0%)	1 (1.4%)
Two doses	2 (3.3%)	0 (0%)	2 (2.8%)
Three doses	37 (60.7%)	0 (0%)	37 (51.4%)
Four doses	16 (26.2%)	10 (90.9%)	26 (36.1%)
Five doses	5 (8.2%)	1 (9.1%)	6 (8.3%)
Primary vaccination			
Adenoviral vector* + mRNA**	13 (21.4%)	3 (27.3%)	16 (22.2%)
Adenoviral vector* x 2	4 (6.6%)	1 (9.1%)	5 (7.0%)
mRNA** x 2	43 (70.4%)	7 (63.6%)	50 (69.4%)
No vaccine	1 (1.6%)	0 (0%)	1 (1.4%)
Vaccine dose 3			
mRNA**	58 (95.1%)	11 (100%)	69 (95.8%)
No third dose	3 (4.9%)	0 (0%)	3 (4.2%)
Vaccine dose 4			
mRNA bivalent***	9 (14.7%)	3 (27.2%)	12 (16.7%)
mRNA**	12 (19.7%)	8 (72.8%)	20 (27.8%)
No fourth dose	40 (65.6%)	0 (0%)	40 (55.5%)
Vaccine dose 5			
mRNA bivalent***	4 (6.6%)	1 (9.1%)	5 (7.0%)
mRNA**	1 (1.6%)	0 (0%)	1 (1.4%)
No fifth dose	56 (91.8%)	10 (90.9%)	66 (91.6%)
Time since infection (days)			
Median [Min, Max]	89 [8, 938]	NA	89 [8, 938]
Missing	0 (0%)	11 (100%)	11 (15.3%)

*ChadOx nCoV-19 **mRNA-1273 or BNT162b2 ***mRNA-1273.214, BNT162B2 Bivalent or mRNA-1273.222 (WT/BA.4-5 or WT/BA.1).

NA, Not Applicable.

**Table 2:** Demographics of 976 healthcare workers sampled February 2023.

	1-4 prior infections (N=877)	No prior infection (N=99)	Overall (N=976)
Age			
Median [Min, Max]	52 [24, 78]	56 [24, 75]	53 [24, 78]
Sex			
Female	763 (87.0%)	90 (90.9%)	853 (87.4%)
Male	114 (13.0%)	9 (9.1%)	123 (12.6%)
Vaccine doses			
No Vaccine	24 (2.7%)	0 (0%)	24 (2.5%)
One dose	8 (0.9%)	1 (1.0%)	9 (0.9%)
Two doses	62 (7.1%)	4 (4.0%)	66 (6.8%)
Three doses	403 (46.0%)	43 (43.4%)	446 (45.7%)
Four doses	333 (38.0%)	45 (45.5%)	378 (38.7%)
Five doses	47 (5.3%)	6 (6.1%)	53 (5.4%)
Primary vaccination			
Adenoviral vector* + mRNA**	185 (21.1%)	25 (25.3%)	210 (21.5%)
Adenoviral vector* x 1	1 (0.1%)	0 (0%)	1 (0.1%)
Adenoviral vector* x 2	95 (10.8%)	13 (13.1%)	108 (11.1%)
mRNA** x 1	7 (0.8%)	1 (1.0%)	8 (0.8%)
mRNA** x 2	565 (64.5%)	60 (60.6%)	625 (64.0%)
No vaccine	24 (2.7%)	0 (0%)	24 (2.5%)
Vaccine dose 3			
mRNA bivalent***	2 (0.2%)	0 (0%)	2 (0.2%)
mRNA**	781 (89.1%)	94 (94.9%)	875 (89.7%)
No third dose	94 (10.7%)	5 (5.1%)	99 (10.1%)
Vaccine dose 4			
mRNA bivalent***	187 (21.3%)	26 (26.2%)	213 (21.8%)
mRNA**	193 (22.0%)	25 (25.3%)	218 (22.4%)
No fourth dose	497 (56.7%)	48 (48.5%)	545 (55.8%)
Vaccine dose 5			
mRNA bivalent***	38 (4.4%)	4 (4.0%)	42 (4.3%)
mRNA**	9 (1.0%)	2 (2.0%)	11 (1.1%)
No fifth dose	830 (94.6%)	93 (94.0%)	923 (94.6%)
Time since infection (days)			
Median [Min, Max]	371 [14, 1050]	NA	371 [14, 1050]
Missing****	73 (8.3%)	99 (100%)	172 (17.6%)

*ChadOx nCoV-19 **mRNA-1273 or BNT162b2 ***mRNA-1273.214, BNT162B2 Bivalent or mRNA-1273.222 (WT/BA.4-5 or WT/BA.1) ****73 individuals were assessed or previously infected based on IgG Nucleocapsid seroconversion.NA, Not Applicable.

### Saliva sample collection

The collection and processing of saliva samples followed standardized protocols and the procedure started with the methods that least interfered with saliva production. First, participants were instructed to passively drool in a clean cup for five minutes. Saliva was transferred using a pipette into a 2mL tube. A buccal swab (the soft end of a FLOQSwab, cat nr. CP501CS01, COPAN Diagnostics Inc., United States) was then immersed in saliva briefly accumulated on the tongue, with no/minimal brushing of the oral mucosa. The swab was transferred into a 15 mL Falcon tube with 0.5 mL PBS. The swab was gently pressed against the tube wall to extract saliva and tubes were vortexed for 10 seconds. Next, participants were instructed to place a Salivette^®^ absorbent roll (cat nr: 51.1534, Sarstedt AG & Co. KG, Germany) between the cheek and gum and hold it still for one minute. The roll was then returned to the Salivette^®^ tube, which was centrifuged for 5 minutes at 1000*g*, leaving saliva at the bottom of the tube. A new absorbent roll was then kept in the mouth for three minutes while saliva production was stimulated by chewing on the cotton roll. The roll was then returned to the Salivette^®^ tube, which was centrifuged for 5 minutes at 1000*g*, leaving saliva at the bottom of the tube. To investigate the effect of centrifugation on passive drool samples and buccal swab samples, the samples were first kept at 4°C over the day, and then centrifuged at 400*g* for one minute at the same temperature to segregate any debris. The supernatant was transferred into 50 μL aliquots.

### Nasal secretion sample collection

A nasal swab (the soft end of a FLOQSwab, cat nr. CP501CS01, COPAN Diagnostics Inc., United States) was gently inserted 2 cm into a nostril, twirled for five seconds, and subsequently placed into a separate 15 mL Falcon tube with 1 mL of PBS. The tubes were vortexed for 10 seconds and the solutions aliquoted.

### Serum sample collection

Blood samples were obtained from participants by venipuncture into serum separator tubes (SST II, BD Vacutainer®). All samples were allowed to clot for at least 30 min at room temperature and then centrifuged at 2000*g* for 10 minutes. The supernatant was collected and stored at -80°C within 1-2 hours for later analyses.

### SARS-CoV-2 spike-specific IgA and IgG in serum, saliva and nasal secretions

Serum (dilution 1:50000), nasal secretion (dilution 1:1000) and saliva (dilution 1:100) spike-specific IgA and IgG were quantified by V-PLEX SARS-CoV-2 panel 31 according to the manufacturer´s instructions (Meso Scale Diagnostics (MSD), Maryland, USA). Total IgA concentrations were quantified using Isotyping Panel 1 Human/NHP Kit (MSD) according to the manufacturer´s instructions (Saliva: IgA, 1:10000; IgG, 1:1000. Nasal secretion samples: IgA 1:5000; IgG 1:500). To correct for possible differences in sampling efficacy, ratios between spike-specific Ig in saliva and nasal secretion samples and total IgA concentration in the same sample were calculated. The ratios were multiplied by 10^7 for graphical purposes. Cut-off values for detectable WT spike-specific IgG and IgA in serum were provided by the manufacturer. To set cut-off-values for detectable nasal secretion and saliva spike-specific IgA and IgG, the mean + 3SD of fourteen pre-pandemic nasal secretion samples, and six pre-pandemic saliva samples were used and set at 3.6 AU/mL for nasal IgA, 5.9 AU/mL for saliva IgA, 3.7 AU/mL for nasal IgG and 22.9 AU/mL for saliva IgG. When analysing and comparing Ig-levels between previously SARS-CoV-2 infected and infection naïve participants, nasal secretion and saliva Ig-levels below cut-off were set to cut-off/√2 for graphical and statistical purposes. Only samples with values above Limit of Detection (LoD, set by MSD Diluent 100 only + 2.5 SD, plate specific) in both spike specific IgA and total IgA were included when analysing correlation coefficients.

SIgA was measured as previously described (19). Briefly, nasal secretion and saliva samples were analysed for SIgA using a V-PLEX SARS-CoV-2 panel according to standard protocol with the following modifications: After sample incubation (dilution 1:50, time 2h) and 3x wash in 150 μL/well of 1X MSD Wash buffer, the supplied detection antibody was switched to a monoclonal mouse anti-human SIgA antibody (HP6141, Calbiochem, Sigma-Aldrich) at a final concentration of 5 µg/mL. After 1h incubation, the plates were washed x3 in 150 μL/well of 1X MSD Wash buffer and a SULFO-TAG conjugated goat anti-mouse antibody (#R32AC-5 ska, MSD) was added at a final concentration of 0.5 µg/mL. The final steps were then performed as specified by the manufacturer. A set of samples with expected high SIgA levels were initially analysed and the three samples with the highest IgA signal were pooled and used as calibrator for the standard curve. A four-fold serial dilution was used with two replicates at each of the seven calibrator levels and buffer only added as an additional zero calibrator blank. The calibration curve (plate specific) was established by fitting the signals from the calibrators to a 4-parameter logistic (or sigmoidal dose-response) model with a 1/Y2 weighting in the DISCOVERY WORKBENCH 4.0 Analysis Software (MSD). By correcting for dilution, the final antibody concentrations in undiluted samples were obtained. As for the IgA measurements, ratios between mucosal spike-specific secretory IgA and total mucosal IgA concentration were then calculated. The ratios were multiplied by 10^7 for graphical reasons. All plates were analysed on a MESO SECTOR S600 instrument (MSD), which is an electrochemiluminescence reader measuring the light emitted from the SULFO-TAG. Results for the antibody assays are reported in arbitrary units (AU)/mL.

### Statistical analysis

Continuous variables (antibody levels) are presented as medians and compared using the Mann-Whitney U test. Categorial variables are presented as percentages and compared with the Chi-square test. The significance of correlation was analysed with Spearman’s rank correlation. Difference between two dependent correlation coefficients were analysed with Williams´s test. Coefficient of variation (CV) was calculated using the formula: CV = (standard deviation/mean) * 100. To investigate variant cross-binding capacity of SARS-CoV-2 specific Ig, a ratio between SARS-CoV-2 BA.5 and WT antibody levels was determined. To assess correlations between variant cross-binding capacity and antibody levels, a spearman rank correlation between SARS-CoV-2 BA.5/WT ratio and SARS-CoV-2 WT antibody levels was determined. All statistical analyses were performed in GraphPad Prism version 9.2.0 (GraphPad Software, San Diego, California, USA) or R version 4.3.2. Within R, the following packages were used: tidyverse, psych, table1 and readxl.

## Results

### SARS-CoV-2 spike-specific IgA, SIgA and IgG in saliva and nasal secretions

**Figure 2:**
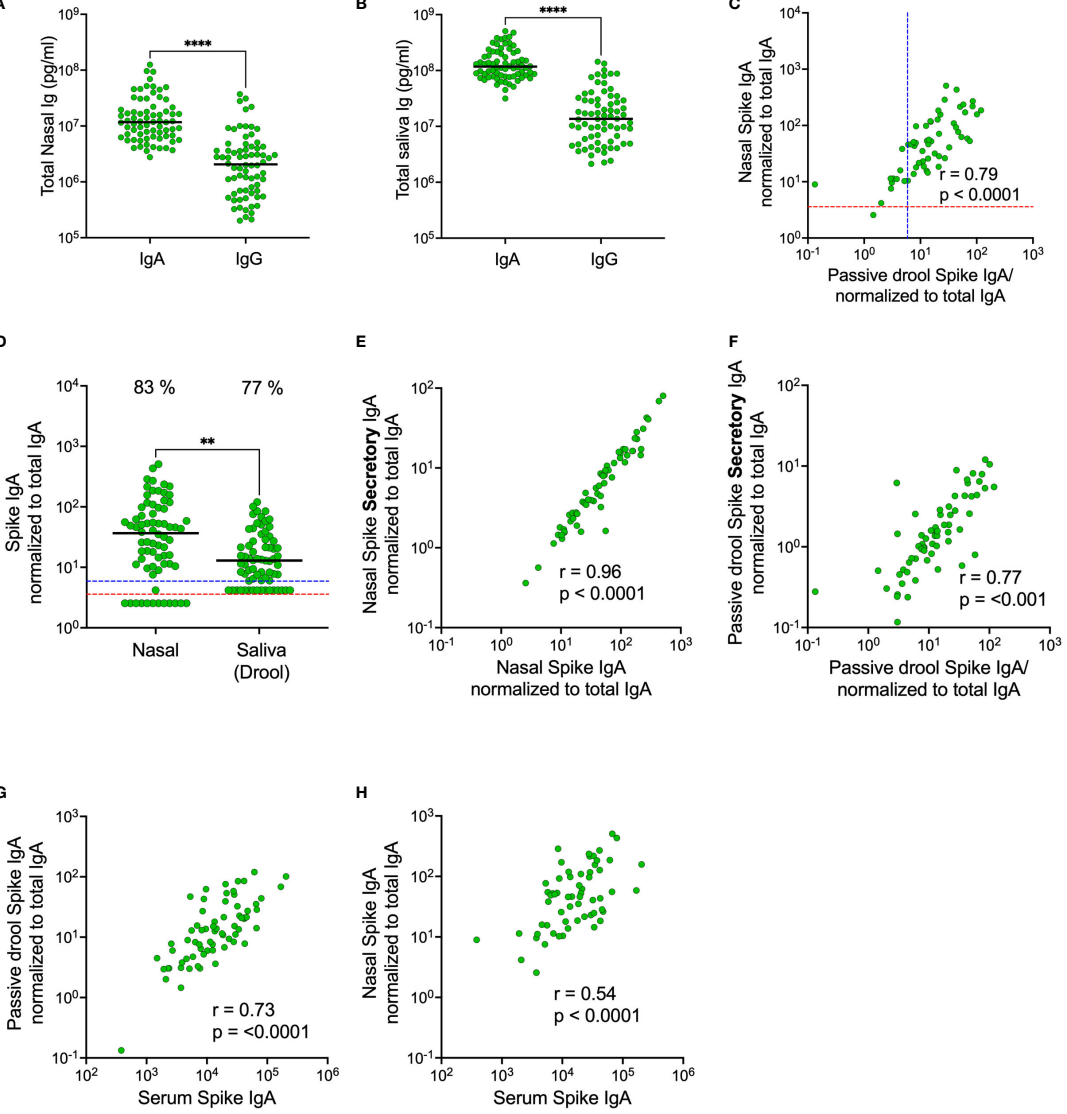
Comparison of total and spike-specific IgA and IgG levels in nasal secretions and saliva (passive drool). Total IgA and IgG-levels in nasal secretions **(A)** and in saliva **(B)**. Correlation between spike-specific IgA in nasal secretions and saliva **(C)**. Levels of spike-specific IgA in nasal secretions and saliva **(D)**. Correlations between spike-specific SIgA and spike-specific IgA in nasal secretions **(E)** and saliva **(F)**. Correlations between spike-specific IgA in serum and saliva **(G)** and serum and nasal secretions **(H)**. Nasal secretion and saliva spike-specific IgA, SIgA and IgG titers were normalized to total IgA and IgG in the same sample. Dashed lines indicate cut-off levels for spike-specific IgA in nasal secretions (red) and saliva (blue). Solid black lines indicate median antibody levels. The percentage of samples above cut-off level is displayed over each scatter plot. **p < 0.01, ****p < 0.0001. r = Spearman’s rank correlation. SIgA, secretory IgA.

We first set out to compare total and spike-specific IgA and IgG in paired nasal secretion, saliva and serum samples from 72 HCW. Samples were collected in November 2022, and the majority of participants had received at least three SARS-CoV-2 vaccine doses (96%) and also experienced at least one prior infection (85%) (**Table 1**). Nasal secretions were collected by swabbing the lining of the anterior nostrils and saliva was collected by passive drooling. Substantially higher levels of total IgA as compared to total IgG were detected in both saliva (nine-fold higher with a median of 1.2 x 10^8^ vs. 1.4 x 10^7^ pg/mL, p<0.0001) and nasal secretions (six-fold higher with a median of 1.2 x 10^7^ vs. 2.1 x 10^6^ pg/mL, p<0.0001) (**Figures 2A, B**). Spike-specific IgA correlated well in nasal secretions and saliva (r=0.79, p<0.0001) (**Figure 2C**). However, spike-specific IgA levels were three-fold higher in nasal secretions as compared to in saliva (median of 36.7 vs. 12.9 AU/mL) (**Figure 2D**). A threshold for detectable spike-specific IgA was set in both nasal secretions and saliva using pre-pandemic nasal secretion and saliva samples. The median spike-specific IgA level was ten-fold above the threshold of 3.6 AU/mL in nasal secretions while only two-fold above the threshold of 5.9 AU/mL in saliva, and there was a trend towards a larger proportion of participants with detectable spike-specific IgA in nasal secretions as compared to in saliva (83% vs. 77%, p = 0.33) (**Figure 2D**).

Considering the possibility of a monomeric IgA transudation from the circulation to mucosal secretions, we determined the proportion of spike-specific IgA bound to the secretory component (secretory IgA; SIgA) in nasal secretions and in saliva. As expected, there were strong correlations between the total population of spike-specific IgA and spike-specific SIgA in both compartments (nasal secretions r=0.96, p<0.001; saliva 0.77, p<0.001), but the correlation was significantly stronger in nasal secretions as compared to in saliva (p<0.0001), suggesting that there may be a larger transudation of monomeric IgA to saliva than to nasal secretions (**Figures 2E, F**). Interestingly, spike-specific IgA in serum correlated stronger (p<0.0001) to the total population of spike-specific IgA in saliva as compared to the total population of spike-specific IgA in nasal secretions (r=0.73, p<0.001 vs r=0.54, p<0.001) (**Figures 2G, H**). Collectively, these findings imply that, although spike-specific IgA correlated well between nasals secretions and saliva, the levels are higher and likely holds a larger proportion of polymeric SIgA in nasal secretions as opposed to slightly lower levels with a larger proportion of monomeric IgA transudate from the circulation, in saliva.

### Secretory IgA has a higher variant cross-binding capacity as compared to monomeric IgA

**Figure 3:**
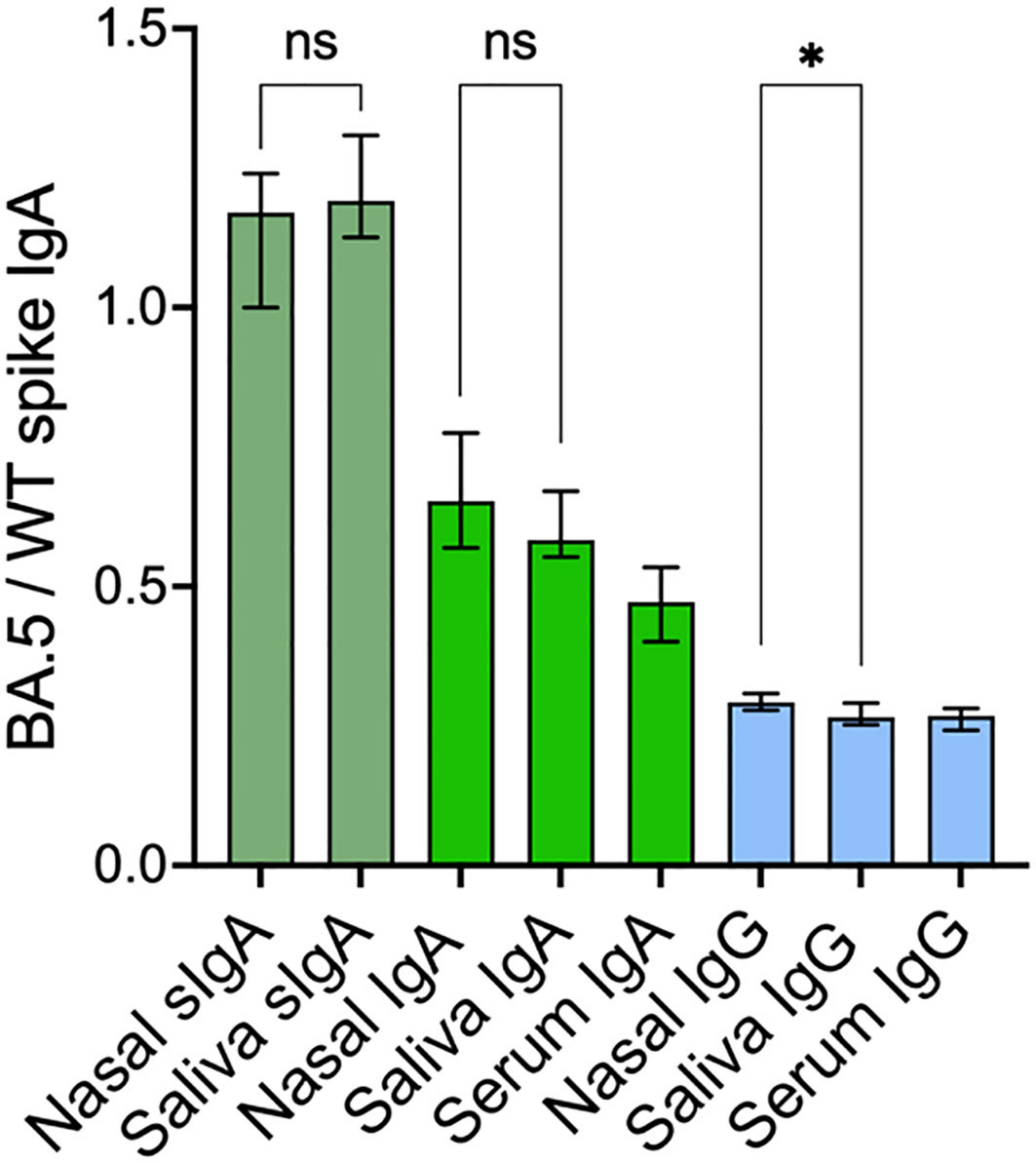
SARS-CoV-2 cross-variant binding capacity of spike-specific SIgA, IgA and IgG. Nasal secretion and saliva spike-specific SIgA, IgA and IgG titers were normalized to total IgA and IgG in the same sample. Green bars represent spike-specific SIgA and IgA and blue bars represent spike-specific IgG. Solid bars represent median ratios, error bars represent 95% confidence intervals. SIgA, secretory IgA; WT, Wild-type. *p < 0.05, ns, non-significant.

Prior studies have demonstrated the superior neutralizing capacity of dimeric IgA over monomeric IgA and IgG (12, 13), potentially due to the polymeric conformation. We therefore proceeded to compare the variant cross-binding capacity of spike-specific SIgA and the total population of spike-specific IgA in nasal secretions and in saliva. The variant cross-binding capacity was assessed by determining the ratio between SARS-CoV-2 BA.5 and wild-type (WT) spike-specific binding for SIgA, IgA, and IgG across compartments. The median ratios between BA.5 and WT spike-specific binding were higher for SIgA in both nasal secretions and saliva (1.17 and 1.19 respectively) as compared to IgA in the same samples (ratio 0.66 and 0.59 respectively), p<0.0001. IgA in serum displayed even lower variant cross-binding capacity (ratio 0.47), p<0.0001, albeit significantly higher than that for IgG in nasal secretion, saliva and serum (ratio 0.29, 0.26 and 0.26 respectively), p<0.0001 (**Figure 3**).

There were only modest correlations between WT spike-specific titers and variant cross-binding capacity for SIgA in nasal secretions (r=0.29, p=0.01) and for IgG in serum (r=0.35, p=0.002), but no other significant correlations were observed between the titers of respective antibody and its variant cross-binding capacity in either of the compartments (all p > 0.05). There were very minor or no differences in the variant cross-binding capacity of each antibody isotype between nasal secretions and saliva, (**Figure 3**), implying that the variant cross-binding capacity is isotype-dependent.

### Infection elicits stronger spike-specific IgA responses in nasal secretions compared to in saliva

**Figure 4:**
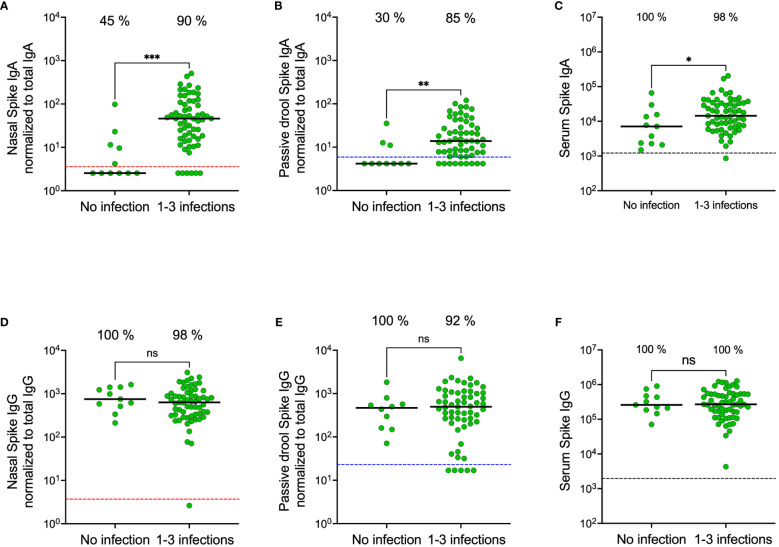
Nasal secretion, saliva (passive drool) and serum spike-specific IgA and IgG levels in participants stratified on prior SARS-CoV-2 infection. Nasal secretion, saliva, and serum spike-specific IgA levels stratified by prior infection **(A–C)**. Nasal secretion, saliva (passive drool), and serum spike-specific IgG levels stratified by prior infection **(D–F)**. Dashed lines indicate cut-off levels for detectable spike-specific Ig in nasal secretions (red), saliva (blue) and serum (black). Solid black lines indicate median antibody levels. The percentage of samples with spike-specific Ig above cut-off level is displayed over each scatter plot. Nasal secretion and saliva spike-specific IgA and IgG titers were normalized to total IgA and IgG in the same sample. Ns, non-significant; *p < 0.05, **p < 0.01, ***p < 0.001.

We recently demonstrated that SARS-CoV-2 infection elicits high and durable levels of spike-specific IgA in nasal secretions (19), but that current systemically administered SARS-CoV-2 vaccines do not trigger a mucosal spike-specific IgA response (18). As expected, and in line with these findings, spike-specific IgA levels were significantly higher in both nasal secretions and saliva from previously infected participants compared to from SARS-CoV-2 infection naïve participants (all p<0.01) (**Figures 4A, B**). Notably, however, these differences were substantially larger in nasal secretions as opposed to in saliva. Spike-specific IgA levels were eighteen-fold higher in nasal secretions from participants with prior infection as compared to SARS-CoV-2 infection naïve participants (median 46.0 vs. 2.5 AU/mL, p<0.001), while corresponding difference was only three-fold in saliva (13.8 vs. 4.2 AU/mL, p<0.01) and two-fold in serum (14 400 vs. 7 300 AU/mL) from the same individuals (**Figures 4A–C**). Spike-specific IgG levels were not higher in participants with prior infection in any of the mucosal compartments nor in serum (**Figures 4D–F**).

### Validation in a larger cohort

**Figure 5:**
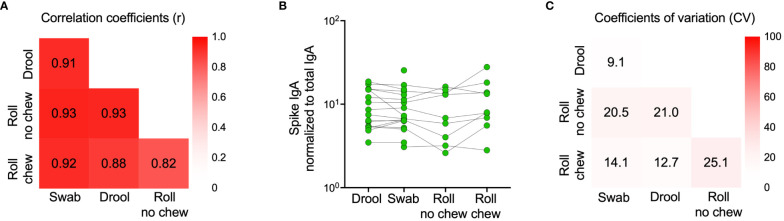
Comparison of spike-specific IgA levels in saliva collected by different methods. Drool (n=16) - Participants were instructed to passively drool into a clean cup for 5 minutes which was then transferred using a pipette into a 2 mL tube. Swab (n=18) - A buccal swab was immersed in saliva accumulated on the tongue, minimizing contact with the oral mucosa and then transferred into a 15 mL Falcon tube with 0.5 mL PBS. Roll no chew (n=9) - An absorbent cotton roll was kept still between the cheek and gum for one minute, the roll was then transferred to a tube provided by the manufacturer and centrifuged for 5 minutes at 1000*g*, leaving saliva at the bottom of the tube. Roll chew (n=9) - An absorbent cotton roll was kept in the mouth for three minutes, during which saliva production was stimulated by chewing, the roll was then transferred to a tube provided by the manufacturer and centrifuged for 5 minutes at 1000*g*, leaving saliva at the bottom of the tube. Correlation coefficients (Spearman’s rank correlations) between sample types are indicated by numbers in the heatmap **(A)**. Individual spike-specific IgA levels in saliva (n=18) **(B)**. Coefficients of variation (CV) between sample types are indicated by percentages in the heatmap **(C)** Spike-specific IgA titers were normalized to total IgA in the same sample.

We next proceeded to validate our findings in a larger cohort of 976 participants sampled in February 2023 (**Table 2**). Due to the time-consuming nature of passive drooling and the relatively large sample size, we first compared this method with the relatively easier saliva collection methods using a cotton roll (Salivette®) with or without chewing or a simple buccal swab in a subset of 18 participants, all with prior infection. Paired analyses revealed strong correlations and comparable spike-specific IgA levels in saliva samples collected by all four methods (**Figures 5A, B**). Furthermore, the coefficients of variation (CV) of spike-specific IgA were low between the collection methods, with the lowest CV between passive drooling and buccal swabbing (%CV for passive drooling and buccal swabbing was 9.1; passive drooling and cotton roll with chewing 12.7; passive drooling and cotton roll without chewing 21.0; buccal swabbing and cotton roll with chewing 14.1; buccal swabbing and cotton roll without chewing 20.5, and cotton roll with and without chewing 25.1) (**Figure 5C**). We next investigated the effect of centrifugation prior to analyses of saliva samples collected with buccal swabbing and by passive drooling. Centrifugation had a smaller effect on saliva spike-specific IgA levels in samples collected by buccal swabbing as opposed to by passive drooling (r=0.85, p=0.006, %CV: 7.2 vs. r=0.71, p=0.08, %CV: 18.8). As the cotton roll protocol stipulates a centrifugation step, we did not examine analyses of spike-specific IgA in saliva collected by cotton rolls without centrifugation. These experiments ensured us that any of these saliva collection methods can be used to generate comparable results, and, for simplicity, we therefore proceeded to sample saliva by buccal swabbing without centrifugation.

**Figure 6:**
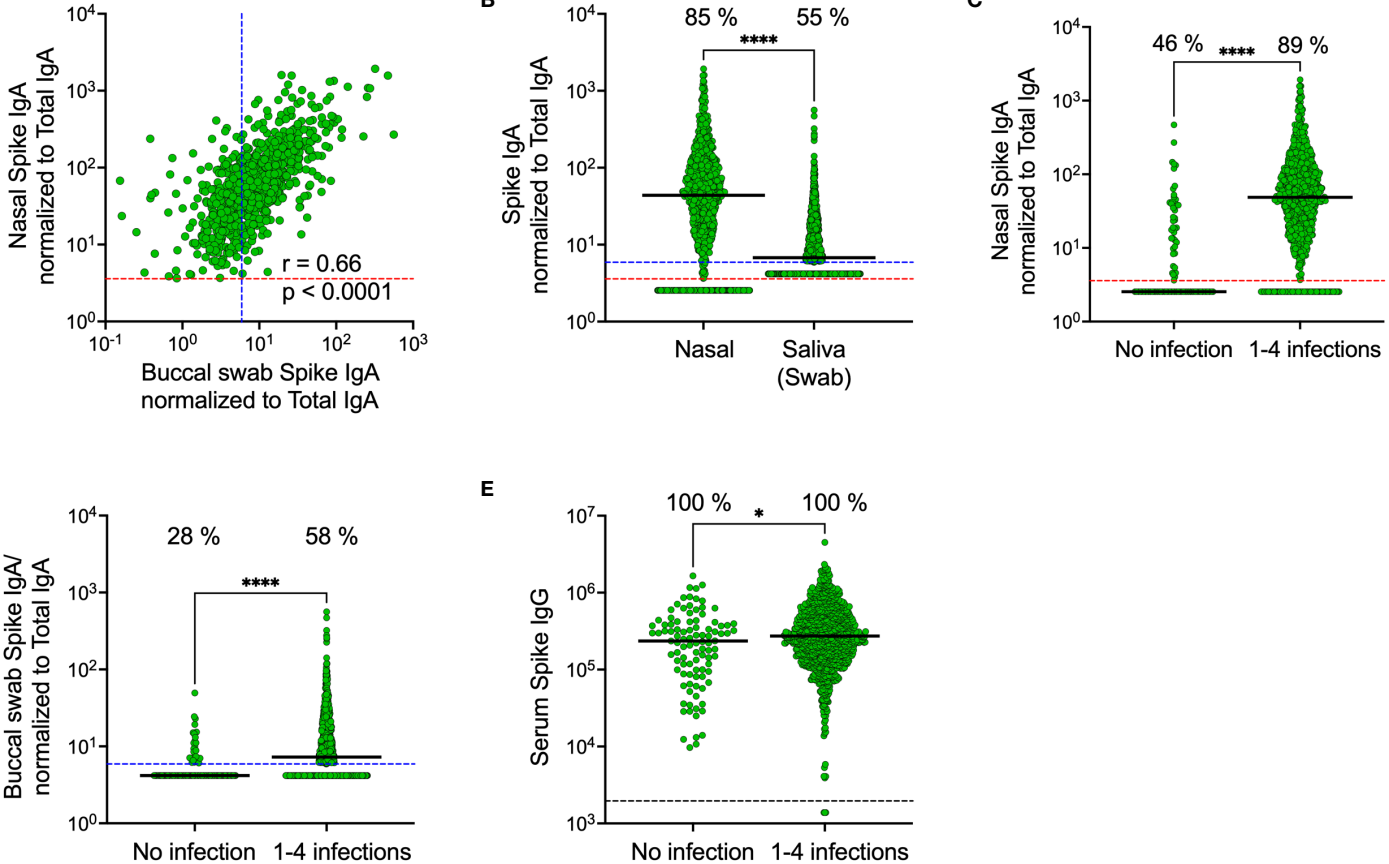
Comparison of SARS-CoV-2 spike-specific IgA levels in nasal secretions, saliva (collected by buccal swabbing) and serum. Correlation between spike-specific IgA in nasal secretions and saliva **(A)**. Spike-specific IgA levels in nasal secretions and saliva **(B)**. Nasal secretion spike-specific IgA levels stratified by prior infection **(C)**. Saliva spike-specific IgA levels stratified by prior infection **(D)**. Serum spike-specific IgG levels stratified by prior infection **(E)**. Dashed lines indicate cut-off levels for detectable spike-specific Ig in nasal secretions (red), saliva (blue) and serum (black). Solid black lines indicate median antibody levels. The percentage of samples with spike-specific Ig above cut-off level is displayed over each scatter plot. Nasal secretion and saliva spike-specific IgA levels were normalized to total IgA in the same sample. Ns, Non-significant; *p < 0.05, ****p < 0.0001. r = Spearman’s rank correlation.

In line with our results from the smaller cohort collected in November 2022, spike-specific IgA levels correlated in saliva and nasal secretions (r = 0.66, p < 0.0001) (**Figure 6A**). Median spike-specific IgA levels were six-fold higher in nasal secretions as compared to in saliva (43.8 vs. 6.8 AU/mL, p<0.0001), a significantly larger proportion of participants had detectable spike-specific IgA in nasal secretions than in paired saliva samples (85% vs. 55%, p<0.0001), and median spike-specific IgA levels were twelve-fold higher than the threshold in nasal secretions (43.8 vs. 3.6 AU/mL) compared to just above cut-off in saliva (6.8 vs 5.9 AU/mL) (**Figure 6B**). Consistent with the findings from the smaller cohort, spike-specific IgA was detected predominantly in nasal secretions and saliva from individuals who had experienced a prior infection, with a larger difference in nasal secretions as opposed to in saliva (median difference between convalescents and naïve was nineteen-fold in nasal secretions, 48.7 vs. 2.5 AU/mL, and two-fold, 7.3 vs. 4.2, AU/mL in saliva) (**Figures 6C, D**). Prior infections had a negligible impact on serum spike-specific IgG levels (**Figure 6E**).

## Discussion

Our findings from two different HCW cohorts, collected at two different time points, and using two different saliva collection methods, clearly demonstrate that SARS-CoV-2 antibody responses differ in the oral and nasal mucosal compartments. Whether our observed higher levels of spike-specific IgA in nasal secretions as compared to in saliva reflects a compartmentalized distribution of IgA secreting cells (38), or stable IgA in nasal secretions in contrast to rapidly waning saliva IgA in an oral environment prone to constant replenishment, warrants further investigation. Regardless of causative mechanism(s), higher levels, along with larger elevations above threshold levels in nasal secretions as compared to in saliva suggest that nasal secretions provide a robust measurement of respiratory mucosal antibody responses, well suited for both clinical trials and larger real-world cohort studies.

The stronger correlations between total spike-specific IgA and spike-specific SIgA in nasal secretions as opposed to in saliva, along with a stronger correlation between serum spike-specific IgA and the total population of spike-specific IgA in saliva than in nasal secretions implies that there may be a, perhaps transient but not negligible, transudation of monomeric IgA from the circulation to saliva. Important to this context, our findings demonstrate a substantially higher variant cross-binding capacity of SIgA as compared to the total population of IgA in both nasal secretions and saliva. The weak or absence of correlations between the levels of respective antibody class and its variant cross-binding capacity in either of the compartments as well as the similarity in variant cross-binding capacity of respective antibody across compartments emphasizes the link between antibody class and variant cross-binding capacity, likely, at least in part, mediated by the structural composition of polymeric IgA. The majority of IgA in both nasal secretions and saliva is SIgA (11, 39), but monomeric IgA may pass to the oral cavity via crevicular fluid (40, 41), which may increase with age and degree of periodontal inflammation and perhaps also be further triggered by saliva collection devices. Our findings thereby emphasize the importance of taking potential impact of flow rate and uncontrolled admixture of serum-derived monomeric IgA into consideration when investigating mucosal immune responses in the oral compartment.

Finally, and in line with recent data (20, 22), nasal and saliva IgA levels were substantially higher in individuals with prior infection compared to SARS-CoV-2 naïve individuals, despite several SARS-CoV-2 vaccine doses. Conversely, there were no significant differences in nasal and saliva IgG levels between previously infected and non-infected individuals, demonstrating how previous infection, but not vaccination, boosts spike-specific mucosal IgA levels. Notably, the differences in saliva IgA levels between previously infected and SARS-CoV-2 naïve individuals were not as prominent as the differences in nasal IgA levels between the same groups. This implies that the detection and quantification of spike-specific IgA in nasal secretions may be a better biomarker for the identification of prior infection than the same analyses in saliva samples.

## Conclusion

Standardization of mucosal sampling protocols allows for data harmonization and comparisons across research studies and clinical trials and are of utmost importance for the evaluation of new mucosal vaccine candidates. Our findings imply an advantage in measuring antigen-specific IgA in nasal secretions as compared to in saliva. We furthermore demonstrate the superior variant cross-binding capacity of SIgA in mucosal secretions, emphasizing the potential protective benefits of a vaccine targeting the upper respiratory tract.

## Data availability statement

The datasets presented in this article are not readily available because of the General Data Protection Regulation. Requests to access the datasets should be directed to charlotte.thalin@ki.se.

## Ethics statement

The studies involving humans were approved by Swedish Ethical Review Authority (dnr 2020-01653). The studies were conducted in accordance with the local legislation and institutional requirements. The participants provided their written informed consent to participate in this study.

## Author contributions

OB: Conceptualization, Data curation, Formal analysis, Investigation, Methodology, Project administration, Visualization, Writing – original draft, Writing – review & editing. KA: Data curation, Formal analysis, Investigation, Methodology, Project administration, Writing – original draft, Writing – review & editing. UM: Data curation, Investigation, Writing – review & editing. MK: Methodology, Project administration, Writing – review & editing. NG: Methodology, Project administration, Writing – review & editing. AS: Funding acquisition, Methodology, Resources, Writing – review & editing. MS: Funding acquisition, Methodology, Resources, Writing – review & editing. JK: Funding acquisition, Investigation, Methodology, Writing – review & editing. KB: Funding acquisition, Methodology, Supervision, Writing – review & editing. MR: Investigation, Methodology, Supervision, Writing – review & editing. SH: Investigation, Methodology, Project administration, Supervision, Writing – review & editing. CT: Conceptualization, Data curation, Formal analysis, Funding acquisition, Investigation, Methodology, Resources, Supervision, Visualization, Writing – original draft, Writing – review & editing. MÅ: Conceptualization, Data curation, Formal Analysis, Funding acquisition, Investigation, Methodology, Resources, Supervision, Visualization, Writing – original draft, Writing – review & editing.
